# Same Session Validation of a Custom‐Built 22G With the Commercial 25G System for EUS‐Guided Portal Pressure Gradient Measurement

**DOI:** 10.1002/ueg2.70194

**Published:** 2026-02-26

**Authors:** Michael Praktiknjo, Nancy Farouk, Zeyu Wang, Juliana Stadtmann, Dominik van de Loo, Markus Kimmann, Carina Gunia, Jörn Arne Meier, Frank Erhard Uschner, Kai‐Henrik Peiffer, Wim Laleman, Jonel Trebicka, Barbara Braden

**Affiliations:** ^1^ Department of Medicine B University Hospital Münster Münster Germany; ^2^ Department of Gastroenterology and Hepatology Section of Liver and Biliopancreatic Disorders and Liver Transplantation University Hospitals Leuven KU Leuven Leuven Belgium; ^3^ European Foundation for the Study of Chronic Liver Failure Barcelona Spain

**Keywords:** endoscopic ultrasound, EUS‐PPG, portal hypertension

## Abstract

**Background and Aims:**

Endoscopic ultrasound‐guided portal pressure gradient (EUS‐PPG) measurement is a promising alternative to hepatic venous pressure gradient (HVPG) assessment, especially in settings where HVPG is unavailable or limited. The commercial 25‐gauge (25G) system showed good correlation with the hepatic venous pressure gradient (HVPG). However, the 25G has drawbacks due to its small caliber and the proprietary pressure transducer. The aim of this study was to validate a custom‐built 22G conventional intravascular pressure transducer system (22G EUS‐PPG).

**Methods:**

In this prospective cohort study, 26 patients underwent EUS‐PPG measurement using both systems during the same session. The primary outcome was the correlation of PPG values. Secondary outcomes included the correlation and variability of portal vein pressure (PVP) and hepatic vein pressure (HVP) measured by both systems.

**Results:**

PPG values showed excellent correlation of both systems (*r* = 0.901, *p* < 0.001). 25G EUS‐PPG correctly identified clinically significant portal hypertension (CSPH, defined as PPG ≥ 10mmHg) in 25 of 26 (96.2%) cases. Portal vein and hepatic vein pressures also correlated significantly (*r* = 0.776 and *r* = 0.673, respectively) between both systems. Variability within both systems was very low to low.

**Conclusion:**

EUS‐PPG measurements obtained using the commercial 25G and custom‐built 22G EUS‐PPG systems were validated. The custom‐built 22G system excels due to pressure‐tracing based quality control, availability and cost‐efficiency.

## Key Summary

- Summarize the established knowledge on this subject
  ◦ EUS‐guided portal pressure gradient (EUS‐PPG) measurement using a commercial 25G system is a minimally invasive alternative to hepatic venous pressure gradient (HVPG) for evaluating portal hypertension.
  ◦ The commercial 25G device lacks real‐time pressure tracing due to a propietary transducer system.
  ◦ Limited availability and reimbursement of this device restrict the clinical adoption of EUS‐PPG in many healthcare systems.
  ◦ No adaptable alternative has previously been validated against the commercial system in a same‐session setting.
- What are the significant and/or new findings of this study?
  ◦ This is the first prospective same‐session validation of a custom‐built 22G EUS‐PPG system against a commercial 25G device under identical conditions.
  ◦ The 22G system showed excellent correlation with the commercial device and equivalent diagnostic performance for clinically significant portal hypertension.
  ◦ Integration with a standard intravascular pressure transducer enables real‐time pressure tracing and broader technical flexibility.
  ◦ The system provides a reliable, safe and cost‐efficient alternative that can expand access to EUS‐PPG across diverse healthcare environments.

## 1 Introduction

Portal hypertension is the main driver of complications of liver cirrhosis, resulting in a significant health care burden [1]. The presence of significant portal hypertension (CSPH) defined as hepatic venous pressure gradient (HVPG) ≥ 10 mmHg stratifies patients at high‐risk for acute decompensation of liver cirrhosis [2]. Thus, identification of CSPH plays a crucial role in the management of chronic liver diseases by indicating treatment decisions such as the initiation of non‐selective beta‐blockers [3].

The HVPG is considered the gold standard for assessing portal pressure [4]. However, HVPG often requires access to interventional radiology, which limits its availability, and it cannot detect presinusoidal portal hypertension. This is particularly relevant in conditions, such as metabolic dysfunction‐associated steatotic liver disease (MASLD), primary biliary cholangitis (PBC), or porto‐sinusoidal vascular disorder (PSVD), where HVPG underestimates portal hypertension systematically [5, 6]. Given that MASLD is becoming the most common cause of chronic liver disease globally, and already is in some regions, the limitations of HVPG are clinically significant.

Endoscopic ultrasound‐guided portal pressure gradient (EUS‐PPG) measurement has gained attention in recent years as a complementary method to HVPG. It allows direct access to the portal and hepatic veins via an endoscopic route, not requiring fluoroscopy or central venous access and thus overcomes some of HVPG's limitations [7].

So far, one dedicated EUS‐PPG platform is commercially available (EchoTip Insight, Cook Medical, Limerick, Ireland). This system uses a 25G FNA needle with a proprietary pressure transducer, which displays a digital reading of the pressure but does not provide real‐time pressure curve tracing. The ENCOUNTER study demonstrated a good correlation of EUS‐PPG with this 25G system and HVPG measurements [8].

Another study also showed good correlation of HVPG with EUS‐PPG but measured with a custom‐built 22G FNA needle system, linked to a standard invasive pressure monitoring system as it is commonly used in intensive care units [9].

The 22G EUS‐PPG system may offer some advantages: Firstly, it allows real‐time display of pressure curves that may help facilitate quantifying the quality of the pressure readings similar to the pressure curve readings in HVPG [4, 10]. Secondly, the standard pressure monitoring systems are calibrated regularly to ensure correct measurements. Thirdly, the thicker 22G FNA needle is less prone to bending, thus providing a more stable puncture trajectory in difficult positions and offering the potential for simultaneous blood sampling from the portal venous compartment. Fourth, the 22G system uses a standard 22G FNA needle that is available at significantly lower costs than the commercial 25G system.

To date, there is no validation of the commercial 25G system with the custom‐built 22G system for EUS‐PPG measurements.

Therefore, the aim of this study was to evaluate the validity of EUS‐PPG measurements obtained by the custom‐built 22G system using a standard intravascular pressure transducer (22G EUS‐PPG) with the commercial 25G system (25G EUS‐PPG) during the same session in the same patients under identical procedural conditions.

## 2 Materials and Methods

### 2.1 Study Design

This is a prospective, single‐center study conducted at University Hospital Münster to evaluate the correlation between EUS‐PPG measurements obtained using a commercial 25‐gauge (25G EUS‐PPG) and 22‐gauge FNA needle systems attached to a standard pressure transducer and reading monitor (22G EUS‐PPG) in the same patients during the same session. The study protocol was approved by the institutional ethics committee (Ärztekammer Westfalen‐Lippe, EC number 2022‐560‐b‐S, Amendment 03), and all participants provided written informed consent.

### 2.2 Study Cohort

Consecutive patients between January and March 2025 with a clinical indication for EUS‐PPG measurement were included in the study. Patients included were all adults (≥ 18 years) with indication for liver evaluation including portal hypertension undergoing EUS‐PPG. Exclusion criteria included pregnancy, severe coagulopathy (INR > 2), thrombocytopenia (platelet count < 50,000/μL), overt clinical signs of portal hypertension (presence of gastroesophageal varices, ascites, spontaneous portosystemic shunts), contraindications to upper GI endoscopy or sedation, or anatomical alterations preventing vascular access via EUS.

The underlying conditions and resulting indications for EUS‐PPG are shown in Table 1.

**TABLE 1:** Categorical values are represented as *n* (% of available data). Continuous values are represented as the median (range).

	Parameter	Entire study cohort (*n* = 26)
General and indication for EUS	Age	51 (20–75)
	Sex (male)	10 (38.5%)
	Indication EUS: Elevated LFT/LTX/Autoimmune liver disease	11 (42.3%)/9 (34.6%)/6 (23.1%)
	Antiplatelet therapy	5 (20.8%)
	Oral anticoagulation	3 (12.5%)
Laboratory values	MELD	8 (6–20)
	White blood cells (10^9^/L)	6.55 (4.15–11.26)
	Hemoglobin (g/L)	13.2 (9.3–18.8)
	Platelets (10^9^/L)	200 (59–413)
	Creatinine (mg/dL)	0.93 (0.6–3.6)
	Sodium (mmol/L)	139 (126–145)
	Bilirubin (mg/dL)	0.6 (0.4–2.0)
	INR	1.04 (0.84–1.42)
	PTT (s)	33 (30–43)
22G EUS‐PPG	Mean PPG	3 (1–20)
	Mean PVP	15 (8–35)
	Mean HVP	11 (4–27)
25G EUS‐PPG	Mean PPG	3 (1–15)
	Mean PVP	18 (6–36)
	Mean HVP	12 (3–27)
Outcomes	Adverse events	0 (0%)

Abbreviations: EUS, endoscopic ultrasound; HVP, hepatic venous pressure; LFT, liver function test; LTX, liver transplantation; MELD, model of end‐stage liver disease; PPG, portal pressure gradient; PVP, portal venous pressure.

### 2.3 EUS Procedure

All procedures were performed by experienced endoscopists (more than 50 prior EUS‐PPG measurements) under sedation using propofol. Patients were positioned in the left lateral decubitus position. The portal and hepatic veins were endosonographically identified via a transgastric approach with the use of color and pulsed wave doppler imaging. For the EUS, linear echoscopes (EG‐3870UTK; Pentax, Tokyo, Japan) and the Arietta V70 platform were used (Hitachi Medical Corp., Tokyo, Japan).

**FIGURE 1:**
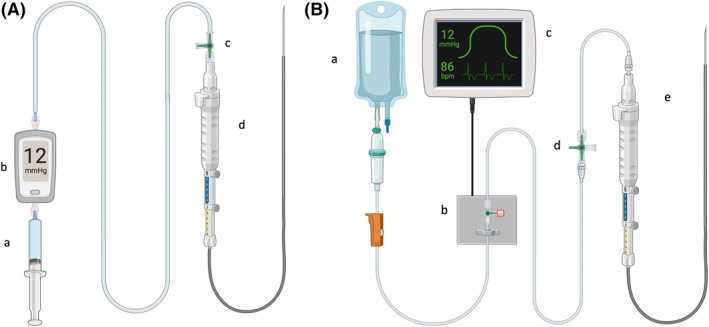
(A) Schematic layout of the commercially available 25G EUS‐PPG system (EchoTip insight). (a) 10mL‐syringe with saline for flushing of the system. (b) Proprietary pressure transducer with digital display showing pressure values in mmHg. (c) 3‐way tap for venting of the system. (d) 25G FNA needle. Created with biorender.com. (B) Schematic layout of the custom‐built 22G EUS‐PPG system. (a) 1000 mL saline in a pressure bag. (b) Pressure transducer with valve for leveling of the system. (c) Monitoring system to display pressure tracings. (d) 3‐way tap for venting of the system. (e) 22G FNA needle. Created with biorender.com.

PPG measurements were first obtained using a commercially available 25‐gauge system (EchoTip Insight, Cook Medical, Limerick, Ireland). With this system, the included proprietary pressure transducer and tubing were used according to the manufacturer's instruction‐for‐use. Before the measurement, the pressure transducer was leveled at the mid‐sternal line to standardize the measurements. After successful puncture of the left intrahepatic branch of the portal vein or left hepatic vein, a stabilization period of at least 60 s was observed before recording of the pressure values from the proprietary digital display (Figure 1A). Portal and hepatic venous pressures were measured three times each, with saline flushings between each reading, to confirm the intravascular position of the needle tip in EUS B‐mode.

After measurements with the 25G system, portal and hepatic venous pressure measurements were repeated three times each, using a 22‐gauge FNA needle (SonoTip ProFlex, Mediglobe‐Endoflex, Rohrdorf Germany). After removal of the stylet, the FNA needle was attached to a pressure reading monitor system (IntelliVue X3, Philips, Amsterdam, Netherlands), normally used for invasive blood pressure measurements (Figure 1B). The pressure transducer was calibrated and leveled at the mid‐sternal line to standardize the measurements. After successful puncture of the left intrahepatic branch of the portal vein or left hepatic vein, a stabilization period of at least 60 s was observed before taking pressure readings. Compared to the commercially available 25G system, this system has a pressure curve tracing (Figure 1B). Thus, a stable pressure curve had to be visible before recording of the pressure values. Portal and hepatic venous pressures were measured in triplets with saline flushing between each reading to confirm the intravascular position of the needle tip in EUS B‐mode.

Both systems were used one after the other in the same patients in the same session under the same procedural conditions.

### 2.4 Outcomes

The primary outcome was the correlation between the EUS‐PPG measurements acquired using the two different systems.

Secondary outcomes were the consistency of portal vein and hepatic vein pressure measurements between 25‐ and 22‐G needles.

Adverse events were recorded as secondary outcomes.

### 2.5 Statistical Analysis

Data analysis was performed using IBM SPSS Statistics, version 29.0.2.0 (IBM Corp., Armonk, NY, USA), and R (R Foundation for Statistical Computing, Vienna, Austria) via RStudio version 2024.12.1 + 563 (Posit Software, PBC).

Continuous variables are shown as medians with ranges, and categorical variables are shown as absolutes and percentages.

We used Pearson's correlation coefficient to analyze the correlations between mean PPG, individual PVP (portal venous pressure), and individual HVP (hepatic venous pressure) values measured with the 25G and 22G needle systems. Bland‐Altman plot was calculated for PPG to assess agreement and systematic biases.

Measurement stability within the 22G and 25G groups was quantified by calculating standardized mean differences between repeated pressure measurements, with values < 0.1 considered negligible.

A *p*‐value < 0.05 was considered statistically significant.

We used the studies comparing the commercial 25G‐system with HVPG, which suggest an effect size *d* of 0.25, 0.87 and 2.87, respectively [8, 9, 11]. The mean effect size was calculated to 1.33. We assumed an even more conservative effect size of *d* = 0.8. The calculated sample size to measure such effects with standard alpha‐error of 0.05 and beta‐error of 0.8 according to common standards was 50 (25 paired measurements).

## 3 Results

### 3.1 General Patient Characteristics

A total of 26 consecutive patients were prospectively included in the study. The median age was 51 years (20–75), and 10 patients (38.5%) were male.

The most common indications for EUS were the evaluation of unknown liver disease (*n* = 11, 42.3%), followed by liver transplant protocol biopsies (*n* = 9, 34.6%) and autoimmune liver diseases (*n* = 6, 23.1%).

Five patients (20.8%) were on antiplatelet therapy (aspirin) at the time of the procedure, and three patients (12.5%) were on oral anticoagulation (apixaban), which was paused 48 h prior to the procedure.

The median MELD score was 8 (6–20). Laboratory values included a median INR of 1.04 (0.84–1.42), a PTT of 33 s (30–43), and a platelet count of 200 × 10^9^/L (59–413).

With the 22G EUS‐PPG system, the median portal vein pressure (PVP) was 15 mmHg (8–35), hepatic vein pressure (HVP) was 11 mmHg (4–27), and the resulting portal pressure gradient (PPG) was 3 mmHg (1–20).

With the 25G EUS‐PPG system, the median PVP was 18 mmHg (6–36), median HVP was 12 mmHg (3–27), and median PPG was 3 mmHg (1–15).

No adverse events were observed during follow up of 6 months (Table 1). No pain medication was required after the procedure in the recovery room or after discharge.

### 3.2 Primary Outcome: Correlation of EUS‐PPG Between 25G and 22G Systems

**FIGURE 2:**
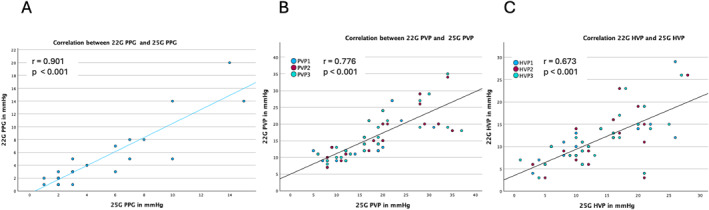
Correlation of portal pressure gradient (PPG) values between 25G and 22G systems. (A) PPG values measured with the 25G system are plotted on the *x*‐axis and those measured with the 22G system on the *y*‐axis. A strong positive correlation was observed (*r* = 0.901, *p* < 0.001). Correlation of portal vein pressure (PVP) and hepatic vein pressure (HVP) values between 25G and 22G systems. (B) Portal vein pressures measured with the 25G system are plotted on the *x*‐axis and those measured with the 22G system on the *y*‐axis (*r* = 0.776, *p* < 0.001). (C) Hepatic vein pressures measured with the 25G system are plotted on the *x*‐axis and those measured with the 22G system on the *y*‐axis (*r* = 0.673, *p* < 0.001).

**FIGURE 3:**
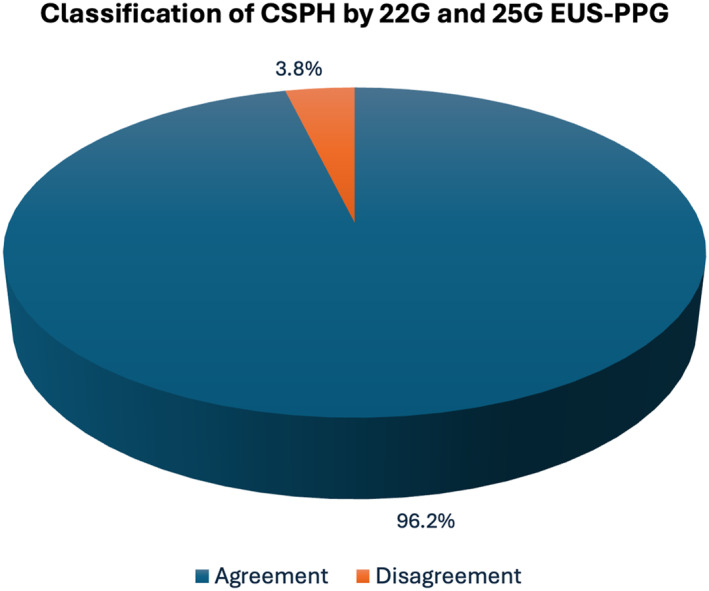
Pie chart of the agreement of the classification of clinically significant portal hypertension (CSPH, defined as EUS‐PPG > 10mmHg) between 25‐ and 22‐G systems. Agreement is plotted in blue and disagreement is plotted in orange.

There was an excellent positive correlation between PPG values measured with the 25G EUS‐PPG and 22G EUS‐PPG systems (*r* = 0.901, *p* < 0.001; Figure 2A). Overall, 9 patients (34%) had portal hypertension (PH) (PPG ≥ 5mmHg) and 2 patients (8%) clinically significant portal hypertension (CSPH) (PPG > 10mmHg). In terms of classification of PH both systems had a perfect agreement (100%). For CSPH, the two systems were concordant in 96.2% of patients and divergent in only 3.8% (95% CI 0.81–0.99) (Figure 3), highlighting excellent agreement between the two systems in classifying patients for PH.

Bland‐Altman analysis showed confirmed excellent agreement of PPG between both systems with narrow limits of agreement (−3.98–4.05) and negligible bias of 0.04 (95%‐CI −0.79–0.87) (Supporting Information S1: Figure S1).

### 3.3 Secondary Outcomes

#### 3.3.1 Correlation of Portal and Hepatic Vein Pressures Between 25G EUS‐PPG and 22G EUS‐PPG Systems

PVP measured using the two systems showed a significant strong positive correlation (*r* = 0.776, *p* < 0.001; Figure 2B). HVPs demonstrated a fairly strong positive correlation (*r* = 0.673, *p* < 0.001; Figure 2C).

### 3.4 Variability of Portal and Hepatic Vein Pressures Between 25G EUS‐PPG and 22G EUS‐PPG Systems

Consistency of measurements within each system was evaluated, standardized mean differences (SMDs) between triplicate measurements of PVP and HVP were calculated (Supporting Information S1: Table S1). The SMDs for portal vein pressure in the 22G EUS‐PPG system were between −0.103 and 0.073. In the 25G EUS‐PPG system, the corresponding SMDs were between −0.243 and 0.162. These findings indicate very low to negligible intrasystem variability in portal vein pressure measurements for both systems. For hepatic vein pressure, the SMDs in the 22G EUS‐PPG system were between −0.184 and 0.308. In the 25G EUS‐PPG system, the SMDs were between −0.227 and −0.056, suggesting slightly higher variability in hepatic vein pressure readings for both systems. The pooled standard deviation was 1.089 mmHg for portal vein measurements and 1.134 mmHg for hepatic vein measurements. Overall, portal vein pressure measurements showed slightly a more consistent reproducibility than hepatic vein pressures across both systems, although absolute differences remained small, indicating good internal reliability.

## 4 Discussion

This prospective study validates the EUS‐PPG measurements obtained using the custom‐built 22G FNA needle and monitoring system against the commercially available 25G EUS‐PPG system. Both systems show an excellent correlation.

While the pressure gradients showed excellent agreement between the two systems, the PVP and HVP were also highly significantly correlated but this was less than excellent for the absolute values. This discrepancy likely results from differences in the pressure leveling methods: the 22G‐EUS PPG system permits repeated calibration to atmospheric pressure, whereas the 25G EUS‐PPG device is leveled only once at the time of activation. The strong consistency of the pressure gradient despite lower agreement in absolute PVP and HVP values supports the use of gradients rather than absolute pressures, consistent with the established HVPG methodology. Furthermore, previous studies have also shown that HVP exhibits greater intrinsic variability compared with PVP due to both technical and physiological factors. Studies in HVPG demonstrated that measurements in the free hepatic vein are highly dependent on the exact position of the measuring catheter tip because of the conical structure of the hepatic vein. When measurements are repeated, variations in catheter positioning can introduce significant bias. These principles naturally also apply to HVP measurements during EUS‐PPG. Moreover, in cases where patients have irregular or small hepatic veins ‐ found regularly in patients with cirrhosis ‐ pressure readings may depend on needle location and potential adherence to the vessel wall. Therefore, a standardization of the EUS‐PPG procedure with harmonized puncture sites, especially for HVP measurements, is needed [12].

Analysis of repeated measurements confirmed high internal consistency of both the commercial and the custom‐built systems, with very low to low variability. Variability was slightly higher for HVP, aligning with known challenges in the measurement of the more dynamic HVP curves. These findings underscore the importance of emphasizing portal pressure gradients when assessing portal hypertension via EUS.

The 22G EUS‐PPG system offers several technical advantages. Continuous visualization of pressure curves enables real‐time validation of measurements, similar to HVPG procedures where pressure curve quality is essential for reliable interpretation [10, 13]. Moreover, recent work emphasized that relying solely on numerical HVPG values without concurrent analysis of the corresponding pressure curves can be misleading. Pressure tracings allow for the identification of artifacts, such as incorrect catheter or needle tip positioning, which may otherwise lead to inaccurate measurements. Continuous monitoring of pressure curves throughout the measurement process is therefore critical to ensure stable and valid readings, particularly when evaluating treatment responses or disease progression in patients with portal hypertension [13]. In addition, the thicker 22G needle demonstrates superior mechanical stability compared to the 25G needle, which is more prone to bending, particularly under the angulated positions frequently required during EUS‐guided liver vascular access [14]. In theory, needle bending may impair pressure transmission and compromise measurement accuracy.

Despite these perceived advantages of the 22G EUS‐PPG system, our study shows that the pressure gradient readings by the commercial 25G EUS‐PPG system are accurate and proves its practicability for the detection of portal hypertension. A potential practical benefit of the 22G EUS‐PPG system is the use of a standard pressure tracing monitor, offering a cost advantage in healthcare systems with limited device coverage. In Germany, the commercial system is priced at the about 1800 Euro, while the disposable components of the custom‐built system cost the about 150 Euro. The cost of the pressure monitoring system is not included. However, due to the sedation, such a system is required in the endoscopy suite anyway. These differences in cost cannot be generalized since health care and reimbursement systems can vary substantially across the world.

Importantly, no adverse events or pain medication use were observed in our study, despite dual puncture of each vessel with both needle systems, respectively. Some studies including meta‐analyses report abdominal pain in up to 11% and 3.6% bleeding [15, 16, 17]. However, these studies mostly included EUS‐guided liver biopsies as concomitant procedures. We attribute our excellent results not only to the relatively small number of patients but also to the large experience of the performing endoscopists with more than 50 EUS‐PPG at the time of this study. Although larger, randomized studies are needed to definitively evaluate safety, the present findings are reassuring and suggest no increased risk associated with the use of both the 25G and 22G needles if performed by experienced endoscopists.

A strength of our study is the tandem design of measuring the pressures in the same patients using both systems in the same EUS session under identical conditions.

Our study did not include invasive pressure measurements via TIPS technique as performed in other studies such as the ENCOUNTER trial [8]. However, EUS‐PPG is mainly intended for compensated stages of liver disease without overt clinical signs of portal hypertension [18]. Therefore, our study included only patients with compensated stages of liver disease and invasive pressure measurements in the TIPS technique cannot be performed ethically in those patients. Even though our sample size is relatively small, it is adequately powered for primary and secondary outcomes. However, subgroup analyses were beyond the scope of this study. Finally, external validation of our results is needed.

In conclusion, EUS‐PPG measurements obtained using the custom‐built 22G EUS‐PPG system were validated against the commercially available 25G EUS‐PPG with excellent agreement. The custom‐built 22G system excels due to pressure‐trace quality control, availability and cost‐efficiency.

**Abbreviations**
CSPH: clinically significant portal hypertension
EC: ethics committee
EUS: endoscopic ultrasound
EUS‐PPG: endoscopic ultrasound‐guided portal pressure gradient
FNA: fine‐needle aspiration
HVP: hepatic vein pressure
HVPG: hepatic venous pressure gradient
IBM: International Business Machines Corporation
IFU: instructions for use
INR: international normalized ratio
MASLD: metabolic dysfunction‐associated steatotic liver disease
MELD: Model for End‐Stage Liver Disease
PBC: primary biliary cholangitis
PPG: portal pressure gradient
PSVD: porto‐sinusoidal vascular disorder
PTT: partial thromboplastin time
PVP: portal vein pressure
SMD: standardized mean difference

## Author Contributions

**Michael Praktiknjo:** conceptualization, methodology, software, data curation, investigation, validation, formal analysis, funding acquisition, visualization, resources, project administration, writing – original draft, writing – review and editing. **Nancy Farouk:** methodology, software, data curation, investigation, validation, supervision, formal analysis, visualization, writing – original draft, writing – review and editing. **Zeyu Wang:** data curation, investigation, formal analysis, visualization, writing – review and editing. **Juliana Stadtmann:** data curation, writing – review and editing. **Dominik van de Loo:** data curation, writing – review and editing. **Markus Kimmann:** data curation, writing – review and editing. **Carina Gunia:** methodology, writing – review and editing. **Jörn Arne Meier:** data curation, writing – review and editing. **Frank Erhard Uschner:** data curation, writing – review and editing. **Kai‐Henrik Peiffer:** data curation, writing – review and editing. **Wim Laleman:** methodology, writing – review and editing. **Jonel Trebicka:** conceptualization, supervision, funding acquisition, visualization, visualization, resources, writing – review and editing. **Barbara Braden:** conceptualization, methodology, investigation, supervision, funding acquisition, visualization, resources, writing – review and editing.

## Conflicts of Interest

J.T. was supported by the German Research Foundation (DFG) project ID 403224013—SFB 1382 (A09), by the German Federal Ministry of Education and Research (BMBF) for the DEEP‐HCC project and by the Hessian Ministry of Higher Education, Research and the Arts (HMWK) for the ENABLE and ACLF‐I cluster projects. The MICROB‐PREDICT (project ID 825694), DECISION (project ID 847949), GALAXY (project ID 668031), LIVERHOPE (project ID 731875), and IHMCSA (project ID 964590) projects have received funding from the European Union's Horizon 2020 research and innovation program. M.P. is funded by the Ernst‐und‐Berta Grimmke Foundation (No. 5/19) and BONFOR research program of the University of Bonn (grant ID 2020‐2A‐07 and 2021‐2A‐07) and by the Deutsche Forschungsgemeinschaft (DFG, German Research Foundation) under Germany's Excellence Strategy—EXC2151‐390873048. He also serves as a consultant for Gore and Cook Medical. W.L. received funding from the MICROB‐PREDICT (project ID 825694), is a co‐holder of the Boston‐Scientific Chair in Interventional Echoendoscopy, and is a consultant for Cook Medical, CSL Behring and MRM Health.

## Supporting information


Supporting Information S1

## Data Availability

Research data are not shared due to the German GDPR.
