# Supplementary material for: Same Session Validation of a Custom‐Built 22G With the Commercial 25G System for EUS‐Guided Portal Pressure Gradient Measurement
Source: United European Gastroenterol J. 2026 Feb 26;14(2):e70194. doi: 10.1002/ueg2.70194 (PMC12945663; doi:10.1002/ueg2.70194)

**Supplementary Table S1:** Standardized mean differences (SMD) between triplicate pressure measurements within 22G EUS-PPG and 25G EUS-PPG systems. PVP measurements showed very low variability. HVP measurements demonstrated slightly higher variability but remained within acceptable limits.

| **Comparison** | **Standardized Mean Difference**  **22G EUS-PPG** | **Standardized Mean Difference**  **25G EUS-PPG** | **Standard**  **Deviation** |
| --- | --- | --- | --- |
| **PVP1 vs. PVP2** | 0.073 | 0.162 | 1.089 |
| **PVP1 vs. PVP3** | -0.053 | 0.020 |  |
| **PVP2 vs. PVP3** | -0.103 | -0.243 |  |
| **HVP1 vs. HVP2** | 0.308 | -0.056 | 1.134 |
| **HVP1 vs. HVP3** | 0.127 | -0.227 |  |
| **HVP2 vs. HVP3** | -0.184 | -0.210 |  |

HVP (hepatic venous pressure), PVP (portal venous pressure), SMD (standardized mean difference)

**Supplementary Figure S1:** Bland-Altman-Plot showing differences in PPG measurements between commercial 25G and custom-built 22G system. CI – confidence interval, LoA – Limits of Agreement.


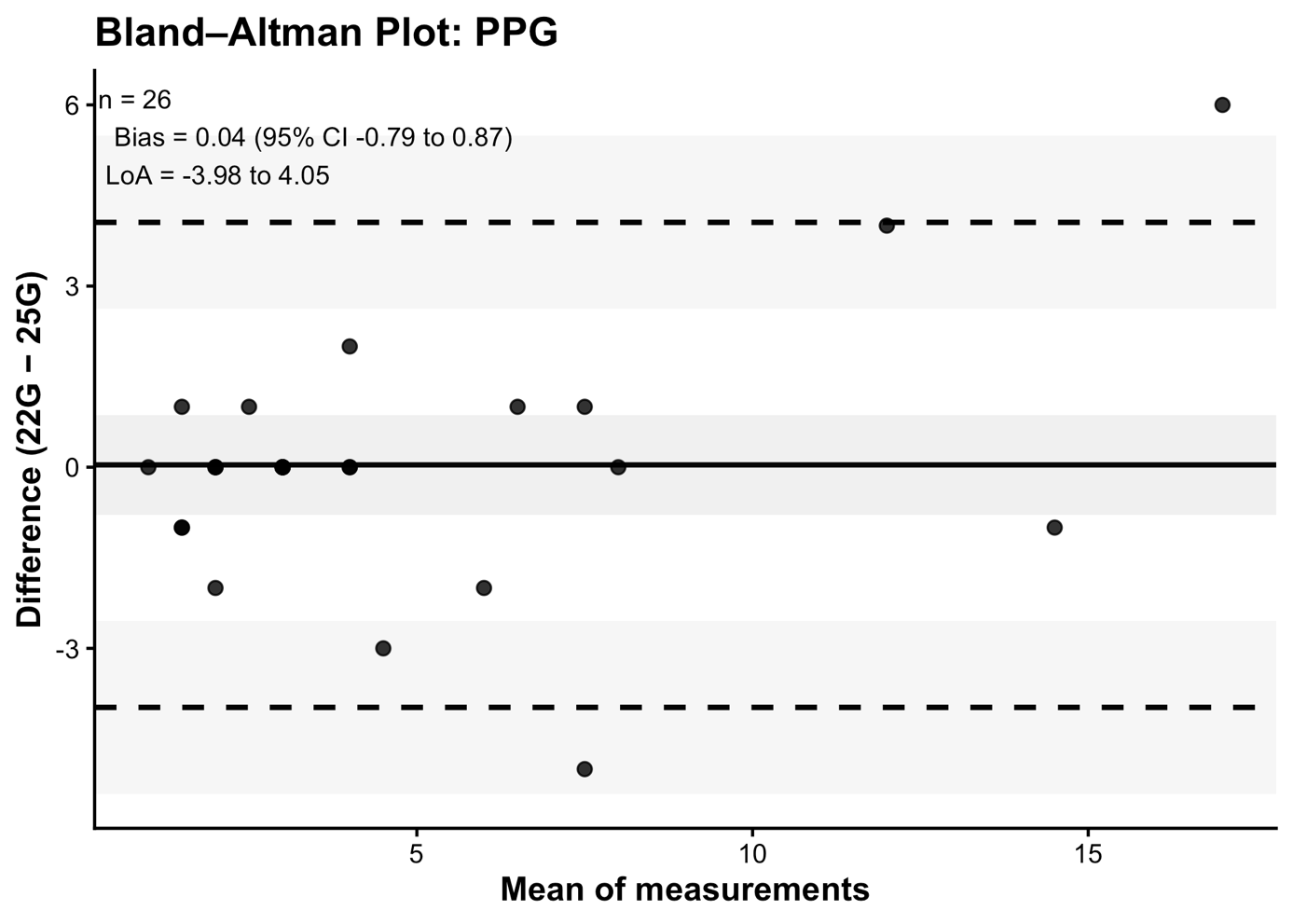

Supplement: Supplementary file 1 — Supporting Information S1 [file UEG2-14-e70194-s001.docx]
